# A superspreading event involving a cluster of 14 coronavirus disease 2019 (COVID-19) infections from a family gathering in Hong Kong Special Administrative Region SAR (China)

**DOI:** 10.5365/wpsar.2020.11.1.012

**Published:** 2020-11-13

**Authors:** Ho Yeung Lam, Tsz Sum Lam, Chi Hong Wong, Wing Hang Lam, Emily Leung Chi Mei, Yonnie Lam Chau Kuen, Winnie Lau Tin Wai, Billy Ho Chi Hin, Ka Hing Wong, Shuk Kwan Chuang

**Affiliations:** a Centre for Health Protection, Department of Health, Hong Kong SAR (China).

## Abstract

**Objective:**

An outbreak of coronavirus disease 2019 (COVID-19) caused by severe acute respiratory syndrome coronavirus 2 (SARS-CoV-2) was first reported in Wuhan, China, in December 2019, with subsequent spread around the world. Hong Kong Special Administrative Region SAR (China) recorded its first confirmed cases on 23 January 2020. In this report, we describe a family cluster of 12 confirmed cases, with two additional confirmed cases from secondary transmission.

**Methods:**

We reported the epidemiological, clinical and laboratory findings of the family cluster, as well as the public health measures instituted.

**Results:**

All 12 confirmed COVID-19 cases were among the 19 attendees of a three-hour Chinese New Year family dinner consisting of hotpot and barbecue dishes. Environmental sampling of the gathering venue was negative. Two additional confirmed cases, who were co-workers of two confirmed cases, were later identified, indicating secondary transmission. Contact tracing, quarantine and environmental disinfection were instituted to contain further spread.

**Discussion:**

Our findings were highly suggestive of a superspreading event during the family gathering. The source was likely one of the cases during the pre-symptomatic phase. The event attested to the high infectivity of SARS-CoV-2 through human-to-human transmission from social activities and argued for the necessity of social distancing in curtailing the disease spread.

An outbreak of coronavirus disease 2019 (COVID-19), caused by severe acute respiratory syndrome coronavirus 2 (SARS-CoV-2), (*1*) was first reported in Wuhan, China, in December 2019. With its spread to other countries and areas, COVID-19 was declared a public health emergency of international concern by the World Health Organization on 30 January 2020.

Current research suggests SARS-CoV-2 to be of zoonotic origin, with the capacity of human-to-human transmission. (*2*) It is highly infectious, (*3*, *4*) and can be transmitted via droplets and contact with contaminated surfaces. Airborne transmission might take place during aerosol-generating procedures. (*5*) Some experts proposed that certain social activities involving water-vapour generation, such as hotpot meals and saunas, were associated with increased risk of transmission. (*6*) Transmission from asymptomatic contacts was also reported. (*7*)

Hong Kong Special Administrative Region SAR (China), a metropolitan city located on China’s southern coast and with intimate economic and social ties with mainland China, reported its first confirmed cases of COVID-19 on 23 January 2020. As of the end February 2020, Hong Kong Special Administrative Region SAR (China) had recorded 95 confirmed cases of COVID-19. Twenty-six cases were local or possibly a locally acquired infection without identifiable sources. In this report, we described a family cluster of 12 confirmed cases, with two additional confirmed cases from secondary transmission.

## Method

### Case identification

Cases of COVID-19 were identified from notification by medical practitioners in Hong Kong Special Administrative Region SAR (China) to the Centre for Health Protection (CHP) under the Department of Health or from contact tracing of confirmed cases.

### Epidemiological investigation

For each notification, CHP initiated a case investigation, including source identification, contact tracing and additional case findings. The incubation period of COVID-19 was defined as 1–14 days before symptom onset. (*8*)

We describe the course of our epidemiological investigation leading to the identification of this family cluster and present the clinical, epidemiological and laboratory findings of the cases.

### Environmental investigation

During the investigation, it was noted that all confirmed cases attended a family gathering before symptom onset. A site visit was conducted to the venue of the gathering with environmental swabs collected for examination.

### Laboratory investigation

All locally confirmed cases of COVID-19 described in this report were laboratory confirmed by the positive detection of SARS-CoV-2 RNA in the patient’s clinical specimens using real-time reverse transcription polymerase chain reaction. The same approach was used for environmental swabs.

### Infection control measures

We describe the various infection control measures instituted.

## Results

### The index case and his family

On 9 February 2020, CHP received notification of a confirmed case of COVID-19 involving a 24-year-old male (Patient 1) who had developed a fever and a productive cough on 30 January 2020. He was admitted to a public hospital on 8 February 2020, and his nasopharyngeal aspirate tested positive for SARS-CoV-2. He did not travel outside Hong Kong Special Administrative Region SAR (China) during the incubation period. He worked as a sales representative and denied having any contact with confirmed COVID-19 cases.

Contact tracing revealed that his parents and maternal grandmother, who resided with him, also had developed symptoms between 28 and 31 January 2020, respectively. They were admitted for isolation and also tested positive for SARS-CoV-2 (Patients 2 to 4).

**Figure 1:**
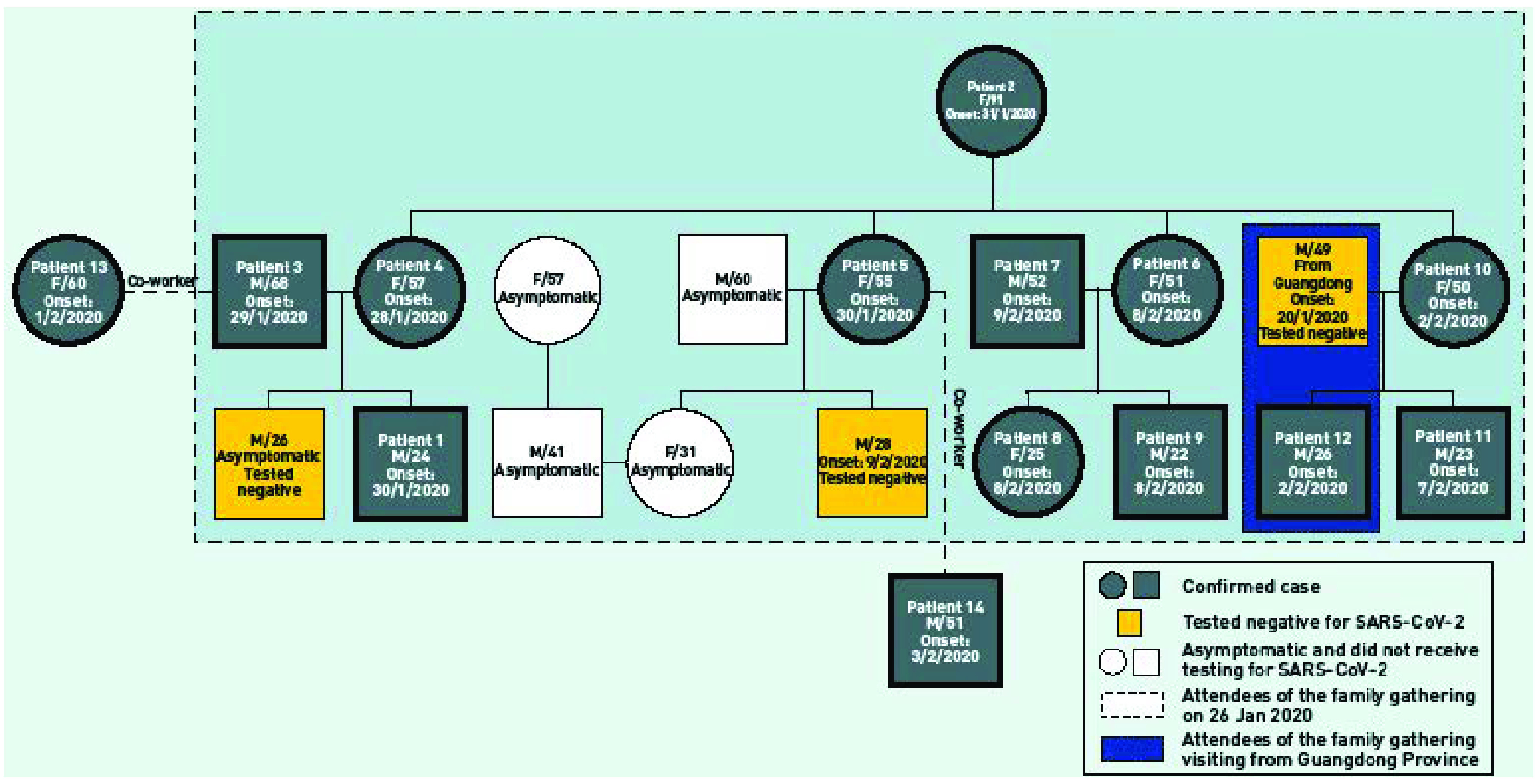
The family tree of the cluster

In view of the proximity of their symptom onset dates, a common source exposure was suspected. Further enquiry revealed that they attended a Chinese New Year gathering with 15 other relatives on 26 January 2020 (**Fig. 1**). At the time of the investigation, eight of them were found to be symptomatic from 30 January to 8 February 2020, and arrangements were made for hospital admission. Seven tested positive for SARS-CoV-2 (Patients 5 to 11). Two of the attendees (a father and his son) were visitors from Guangdong Province, China, and had already returned home at the time of our investigation. We were later informed by the Health Commission of Guangdong Province that the son had developed a cough and runny nose on 2 February 2020 and had tested positive for SARS-CoV-2 on 10 February (Patient 12). The father reported having had a cough for a few days beginning 20 January 2020, which had subsided during the gathering. His respiratory specimen collected on 9 February was negative for SARS-CoV-2. His serology remained negative for SARS-CoV-2 antibodies.

[**insert** Figure 1]

### Family gathering

The 19 attendees lived in several different residences, and the family gathering was the only occasion attended by all 12 confirmed cases during their incubation periods. It was held in a commercial party room during the evening and lasted for about three hours. The attendees had an indoor hotpot dinner and a barbeque held at an outdoor area. No game meat or wild poultry was consumed. They also played mah-jong and snooker. None of the attendees were symptomatic during the gathering. Staff of the party room did not enter the room during the gathering, and there were no other patrons that evening. None of the staff and patrons who used the room in the following days reported symptoms.

### Environmental investigations

A site visit was conducted at the party room on 9 February 2020. Environmental swabs were taken at 18 high-touch areas, including doorknobs, door handles, table surfaces and edges, and light switches. All tested negative for SARS-CoV-2.

### Additional case finding and infection control measures

Extensive contact tracing was conducted for each individual patient confirmed in Hong Kong Special Administrative Region SAR (China). All symptomatic contacts were isolated in a public hospital for treatment and SARS-CoV-2 testing. Asymptomatic contacts were quarantined in quarantine facilities or put under medical surveillance, depending on the nature and duration of contact with the patient. Forty-six close contacts and 166 other contacts were identified. Among them, two contacts who were co-workers of Patients 3 and 5 developed symptoms, one on 1 February and the other on 3 February, and tested positive for SARS-CoV-2 (Patients 13 and 14).

**Figure 2:**
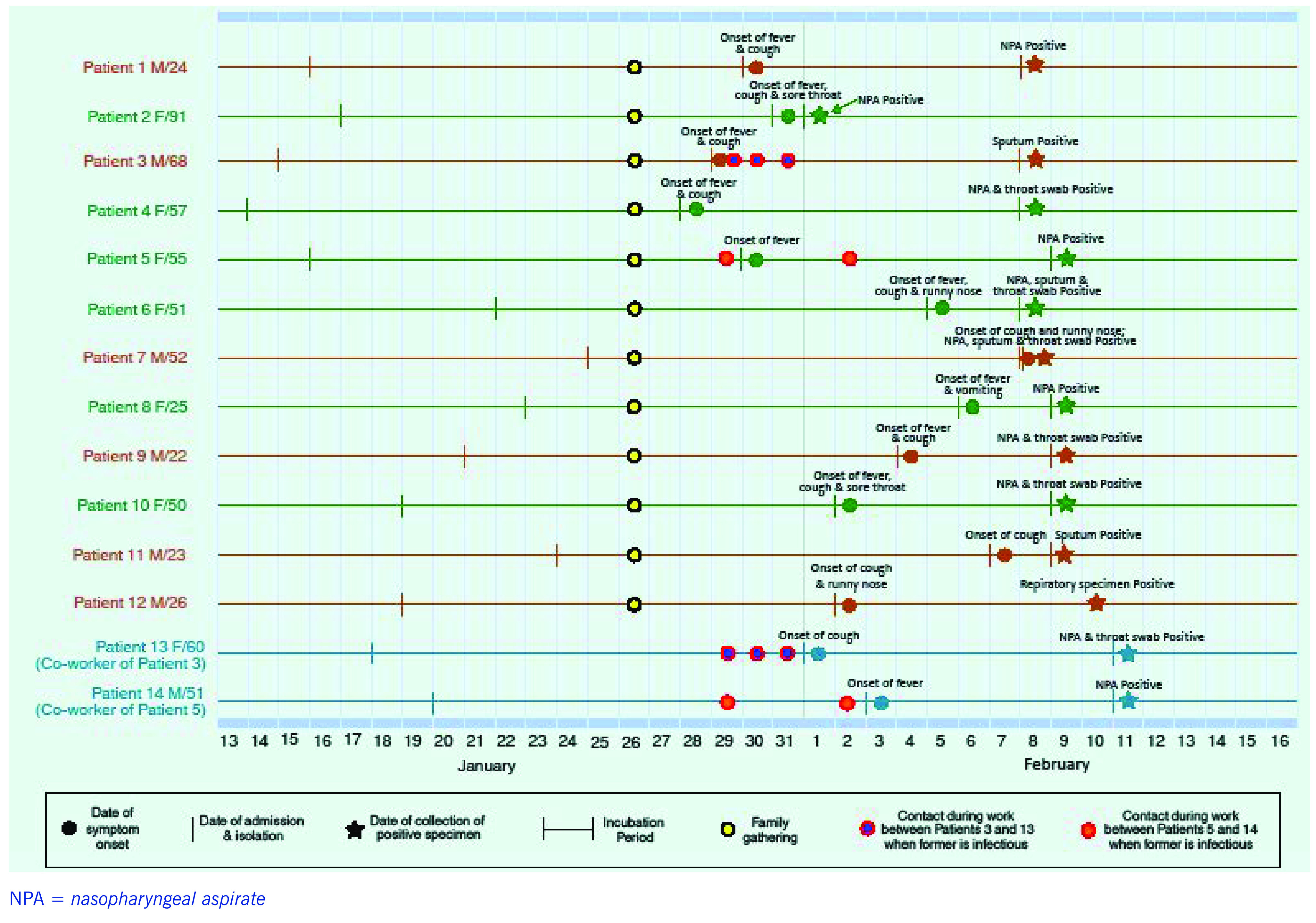
Chart illustrating key events of Patients 1 to 14

Overall, the entire cluster of 14 confirmed cases consisted of seven males and seven females aged 22 to 91 (median: 51). All of them had a record of good health and had no history of travel outside Hong Kong Special Administrative Region SAR (China) during the incubation period, except Patient 12 who had been visiting from China. They all presented with upper respiratory symptoms and/or fever (**Fig. 2**).

[**insert** Figure 2]

Environmental cleansing and disinfection were arranged for the party room, the residences of the cases and their workplaces. As of the end of February, all cases remained stable, and eight (Patients 1, 3, 4, 5, 9, 10, 12 and 13) were discharged. No further cases related to this cluster were identified.

## Discussion

This was the largest COVID-19 family cluster recorded in Hong Kong Special Administrative Region SAR (China) at the time of our reporting. Our epidemiological investigation suggested that primary transmission took place during the family gathering, with secondary transmission leading to the infection of two more cases. As none of the attendees were symptomatic during the gathering, it was likely that pre-symptomatic transmission from one of the attendees had occurred.

Our investigation supported and supplemented the current understanding of the COVID-19 infection. In this cluster, the incubation periods ranged from 2–13 days, which is compatible with the current knowledge. Nevertheless, those with a longer incubation period might represent secondary interfamilial transmission. Our findings also supported human-to-human transmission of SARS-CoV-2. As the family gathering was the only occasion attended by the 12 patients during the incubation periods, it demonstrated the high infectivity of SARS-CoV-2 (as 11 out of 17 susceptible attendees, excluding the potential source, were infected) and its ability to cause a superspreading event.

Environmental factors and behavioural factors have been proposed as risk factors of a superspreading event. (*9*) For example, one study in Japan demonstrated 18.7 times higher odds for transmission in a closed compared with an open-air environment. (*10*) In our cluster, part of the family gathering took place in a party room that was a closed environment. Nevertheless, we were unable to determine the significance of environmental contamination in the transmission chain in this cluster. Moreover, the transmission of SARS-CoV-2 could be enhanced through close and prolonged social contacts without wearing a mask, such as in the family gathering described above.

Although the family gathering involved a hotpot dinner, there was not enough information to support the expert hypothesis that it could enhance SARS-CoV-2 transmission through water-vapour generation.

It is also noted that it took more than one week since symptom onset for most cases in this cluster to receive COVID-19 testing. In fact, several cases had consulted primary care physicians, but they were not tested as tests were only available then in public hospitals and the CHP laboratory. Subsequently, COVID-19 testing had been made available at the primary care level to allow earlier identification of cases in the community. (*11*)

Our investigation had several strengths. Our immediate investigation allowed identification of the possible sources and the establishment of the transmission chain. Extensive contact tracing allowed swift identification of more confirmed cases and ensured contacts were quarantined and put under medical surveillance. The timely institution of these infection control measures allowed complete case ascertainment in this cluster and shed light on the transmission dynamic of COVID-19.

On the other hand, there were some limitations regarding our investigation. Our environmental investigation was conducted two weeks after the family gathering, which might limit the positive yield of the environmental sampling. Cases who remained asymptomatic might not be identified.

In Hong Kong Special Administrative Region SAR (China), family gatherings involving relatives from other extended families and friends are quite common during major festivities (e.g. Chinese New Year) and are considered an important local tradition. These occasions offer good opportunities for superspreading of a highly infectious agent such as SARS-CoV-2.

Social distancing has been advocated as one of the community mitigation measures during influenza pandemics. It entails an increase in physical distances and a reduction of gatherings in dense social settings. (*12*) With the continuing global spread of COVID-19, apart from advocating personal hygiene and protection, social distancing might be necessary to curtail further disease spread in the community, especially for preventing occurrence of superspreading events.
